# Publisher Correction: The Neurocognitive Architecture of Individual Differences in Math Anxiety in Typical Children

**DOI:** 10.1038/s41598-018-28762-7

**Published:** 2018-07-23

**Authors:** Charlotte E. Hartwright, Chung Yen Looi, Francesco Sella, Alberto Inuggi, Flávia Heloísa Santos, Carmen González-Salinas, Jose M. García Santos, Roi Cohen Kadosh, Luis J. Fuentes

**Affiliations:** 1 Aston Brain Centre, School of Life and Health Sciences, Aston University, Birmingham, UK; 2 Department of Experimental Psychology, University of Oxford, Oxford, UK; 3 School of Experimental Psychology, University of Bristol, Bristol, UK; 4 Istituto Italiano di Tecnologia, Genova, Italy; 5 Departamento de Psicología Básica y Metodología, Facultad de Psicología, Universidad de Murcia, Murcia, Spain; 6 Departamento de Psicología Evolutiva y de la Educación, Facultad de Psicología, Universidad de Murcia, Murcia, Spain; 7 Servicio de Radiología, Hospital Morales Meseguer, Murcia, Spain

Correction to: *Scientific Reports* 10.1038/s41598-018-26912-5, published online 31 May 2018

In the original version of this Article, the author Jose M. García Santos was incorrectly indexed. This error has now been corrected.

