# Supplementary material for: The Neurocognitive Architecture of Individual Differences in Math Anxiety in Typical Children
Source: Sci Rep. 2018 May 31;8:8500. doi: 10.1038/s41598-018-26912-5 (PMC5981328; doi:10.1038/s41598-018-26912-5)
Supplement: Supplementary file 1 — Supplmentary Info [file 41598_2018_26912_MOESM1_ESM.pdf]

SUPPLEMENTARY INFORMATION

The Neurocognitive Architecture of Individual Differences in Math Anxiety in Typical Children

Charlotte E Hartwright* [1,2], Chung Yen Looi* [2,3], Francesco Sella [2], Alberto Inuggi [4], Flávia Heloísa Santos [5], Carmen González-Salinas [6], Jose M. García Santos [7], Roi Cohen Kadosh [2] and Luis J Fuentes [5]

* authors contributed equally

Corresponding author: Charlotte E Hartwright

Aston Brain Centre, School of Life and Health Sciences, Aston University, Birmingham, B4 7ET, UK.

Email: c.hartwright@aston.ac.uk | Tel: +44 (0) 121 204 3000

[1] Aston Brain Centre, School of Life and Health Sciences, Aston University, Birmingham, UK

[2] Department of Experimental Psychology, University of Oxford, Oxford, UK

[3] School of Experimental Psychology, University of Bristol, Bristol, UK

[4] Istituto Italiano di Tecnologia, Genova, Italia

[5] Departamento de Psicología Básica y Metodología, Facultad de Psicología, Universidad de Murcia, Murcia, Spain

[6] Departamento de Psicología Evolutiva y de la Educación, Facultad de Psicología, Universidad de Murcia, Murcia, Spain

[7] Servicio de Radiología, Hospital Morales Meseguer, Murcia, Spain

Supplementary Results

Grade	Numerical Cognition									Executive Function (BRIEF)								
	Math Anxiety Scale			Woodcock-Johnson III Achievement			Number line task			Working Memory			Inhibit			Shift		
	n	M	SD	*n*	M	SD	*n*	M	SD	*n*	M	SD	*n*	M	SD	*n*	M	SD
2	8	37.13	14.73	8	85.50	12.59	8	5.50	1.49	-	-	-	-	-	-	-	-	-
3	21	41.29	18.41	21	107.71	13.23	19	6.42	2.16	21	18.52	4.58	21	14.81	4.26	21	12.57	2.93
4	22	38.36	17.98	22	133.82	21.92	19	8.11	1.74	21	15.95	4.63	21	15.55	3.35	21	12.36	3.06
5	16	46.56	15.99	16	144.81	22.38	15	8.17	2.25	16	17.94	4.15	16	15.69	2.82	16	13.25	3.42
6	12	57.08	25.05	12	159.75	23.09	12	8.79	1.62	12	17.92	5.11	12	15.42	3.60	12	12.75	3.05

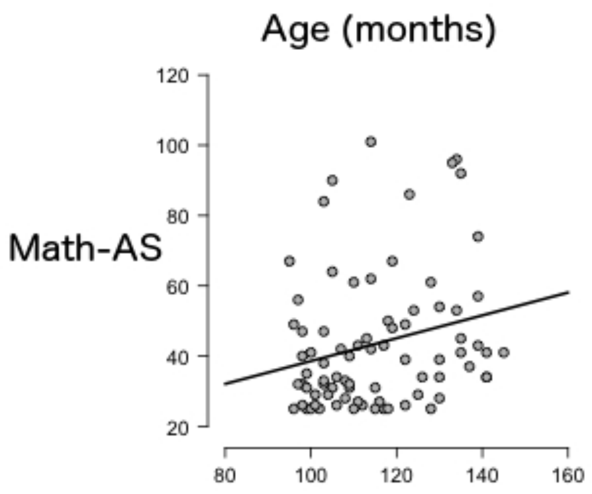

Figure S1. Association between MA and Age. Scatter plots illustrate a weak, positive relationship between MA, age and school grade. Pearson's r 0.237 and 0.283 for age and grade respectively.

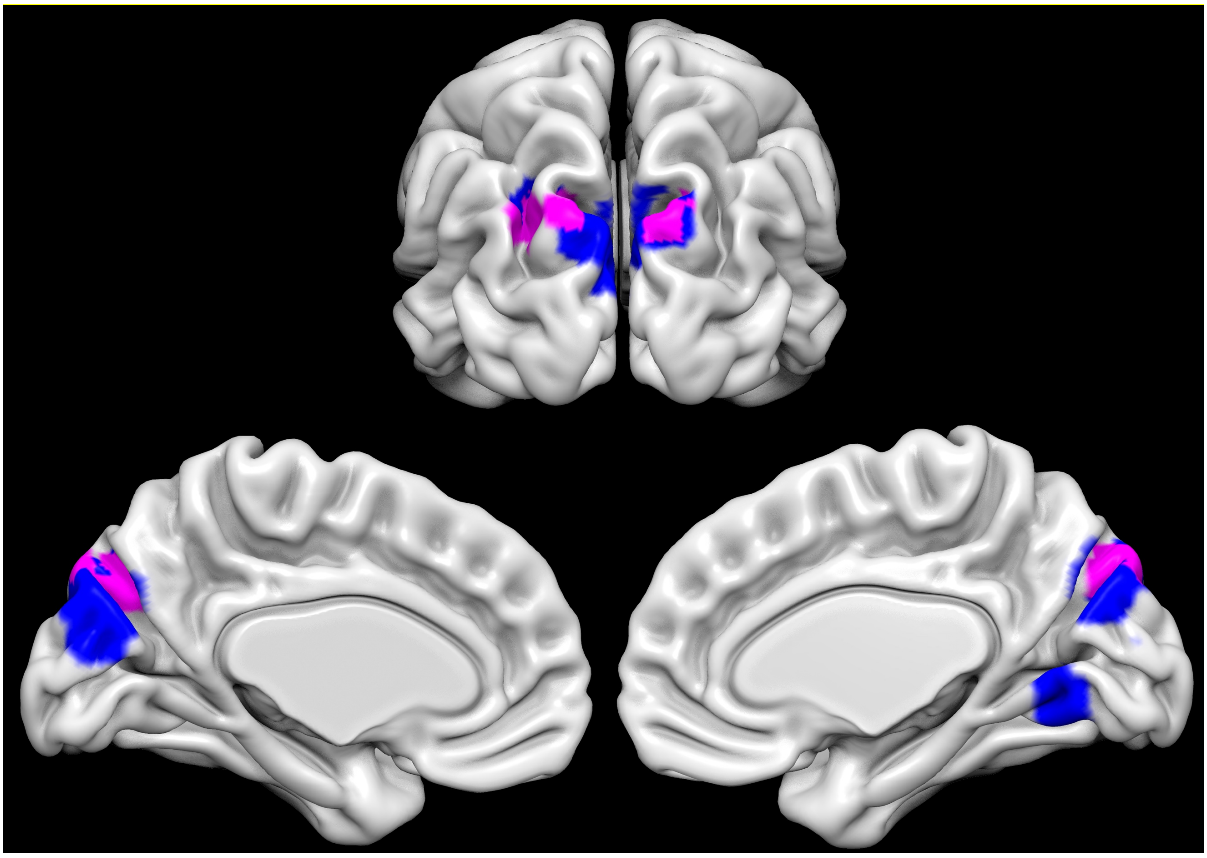

Figure S2. Grey matter correlates of Math Anxiety and Associated Executive Functions. Surface rendered image reflects a spatial map rendered onto a template brain. All coloured areas reflect those grey matter voxels that were significantly negatively associated with MA, controlling for age, recruitment source, handedness and biological sex. The blue cluster map shows those voxels that were previously identified as being negatively associated with MA. This map is spatially identical to that presented in the figure in the main text. The magenta voxels are those voxels that were no longer associated with MA, after controlling for attention, working memory and math achievement. Only grey matter voxels that survived the multiple comparison corrections are shown. All images are presented in neurological convention, where the left of the image reflects the left of the brain. Surface rendering created using Surf Ice (v 10.11.16) [Computer software], available www.nitrc.org/projects/surfice/

Supplementary Methods

Materials

Math Achievement

The Woodcock-Johnson III (WJ-III) Achievement (ACH) battery comprises 4 subtests:

- Calculation test measures performance on simple mathematical computations including addition, subtraction, multiplication and division, which increase in difficulty during the test. It consists of 45 problems of increased complexity with no time limit. The test is terminated by 6 consecutive incorrect answers.
- Math Fluency test measures performance on mathematical operations and fluency in operating with numbers through basic calculus operations such as addition, subtraction, and multiplication facts. It consists of 160 arithmetic problems, and children were instructed to solve as many as possible within a 3-minute time limit.
- Quantitative Concepts measures mathematical knowledge and quantitative reasoning. It is comprised of two subtests: Concepts and Series. The Concept subtest involves counting or identification of numbers, shapes, and sequences, mathematical formulas and terms. It consists of 34 items of increasing difficulty, which were read to the participant. The Series subtest requires the participant to identify a pattern from a series of written numbers and provide the missing number to complete the series. It consists of 23 problems with increasing difficulty.
- Applied Problems test measures quantitative reasoning, mathematical performance, and mathematical knowledge. It requires participants to listen to the problem, identify the procedure to follow and perform simple calculus operations. It involves filtering of appropriate information, and exclude extraneous information. It consists of 62 problems of increasing difficulty, presented orally and visually. This test is terminated by 6 consecutive incorrect answers.

Procedure

Neuroimaging Analyses

The first GLM (main article, Figure 1 and Table 3) comprised the Math-AS scores as an explanatory variable (EV), plus several potentially confounding EVs: recruitment source, age, sex and handedness. Contrasts for 1) positive- and 2) negative-associations between grey matter density (GMD) and MA were run, while controlling for the confounding EVs. Recruitment source was coded using two explanatory variables (EVs) containing an indicator. All other variables were mean-centered across the sample prior to entry into the GLM, and included as individual EVs. In addition to correction for multiple comparisons across the brain, to correct for running both a positive and negative contrast in the GLM, our alpha was set at $p < .025$ (Bonferroni correction, where alpha = .05/2).

To interrogate this initial result, a further series of models were conducted. These were applied to the voxels that were identified as significantly, negatively associated with the Math-AS from the first model. The second GLM replicated the first, but included a further 3 EVs to comprise the mean-centered values for SHIFT, WORKING MEMORY and WJ. As not all participants had completed the BRIEF, an additional 8 nuisance EVs to counter these missing data were also added. From this GLM, a contrast was computed to re-assess the negative association with MA, plus 3 additional contrasts to assess theoretically driven, directional hypotheses for the 3 new measures. Negative contrasts were computed for both SHIFT and WORKING MEMORY, based on the assumption that increased difficulty with attentional control and working memory would reflect reduced GMD, plus a positive contrast for WJ, based on an expected positive association between math achievement and GMD. FSLmaths was used to pinpoint those voxels that were no longer showing a negative, statistically significant association with MA (see *Supplementary Fig. S2*).

As the second GLM comprised 8 fewer participants' data (due to missing data from the BRIEF), a third GLM was computed to verify that this did not result in a reduction in statistical power, as this could offer an alternative explanation for the results of the second GLM. Thus, the first GLM was replicated, and a further 8 nuisance EVs added to reflect these missing data. The contrast for a negative association with

MA was re-run, constrained to the voxels identified in the original analysis. FSLutils was used to compare the resulting cluster map with that determined from the first GLM. This confirmed that there was no reduction in voxel count due to the omission of data from the 8 participants who had not completed the BRIEF.

Additional Information

Data Availability

The research data supporting this publication are available on the Open Science Framework repository, see DOI 10.17605/OSF.IO/PDFJE. The senior author, LJF, may be contacted regarding the wider dataset.
